# Individual and Institutional Factors Preventing Completion of Research by Medical Graduate Students at Cairo University: Questionnaire Study

**DOI:** 10.2196/23235

**Published:** 2021-08-09

**Authors:** Mohammed A Azab, Sharef Fawzy Hasaneen, Ahmed Reda, Ashraf Asous, Ahmed Y Azzam

**Affiliations:** 1 Faculty of Medicine Cairo University Cairo Egypt; 2 Cairo University Hospitals Cairo Egypt; 3 Faculty of Medicine October 6 University 6th October City Egypt

**Keywords:** research, Cairo University, medical, nonpublication

## Abstract

**Background:**

Medical research plays a significant role in advancing the level of health care worldwide. This research is a crucial part of the development of any educational system. In developing countries, the publication rate related to the medical sciences is lower than that in developed countries.

**Objective:**

The aim of this study was to explore the causes of delay in publishing research and the factors that hinder the completion of master’s degree projects in a group of medical graduate students at Cairo University Faculty of Medicine.

**Methods:**

A web-based questionnaire was introduced to approximately 150 medical graduates in different specialties through social media. The questionnaire aimed to investigate the reasons for delays in publishing master’s degree manuscripts after graduation among a group of medical graduates.

**Results:**

Of the graduates contacted, 130 responded to the web-based survey. The ages of the participants ranged from 23-38 years (SD 3.88); 72 of them were male, and 58 were female. Causes of noncompletion of manuscripts were analyzed; lack of proper research training and the absence of supportive mentorship were top reasons. We found a significant relationship between being married and failing to complete the assigned project from its start up to publication. Moreover, we found that the frequency of nonfulfillment increased among those who experienced poor mentorship.

**Conclusions:**

Several factors are contributing to the delay in publication of medical manuscripts related to research projects by medical graduates of the Cairo University Faculty of Medicine. Pensive supervision must be implemented to decipher the persistent institutional problems that obstruct research progress.

## Introduction

Currently, medicine is considered to be not only an art of clinical skill but a multidisciplinary approach that adapts research to clinical achievements. Reflecting on the importance of this consideration, medical schools have started to add research curricula along with medical classes to increase the motivation of students to participate actively in research projects. It is now obligatory for medical students at different medical schools in the United Kingdom to participate in a research project as an essential step for their future medical career [1]. Hypothesis testing and evidence-based medicine are now hallmarks of the medical sciences to ensure excellent care for patients.

Currently, in North America and the United Kingdom, medical program directors ask applicants about their work on research projects when they apply to residency programs, and an applicant has merit if their application contains many cited publications. Although schools invest many resources to improve the research skills of medical students and graduates, a large cohort of these students are not interested in scholarly activities. It is important to evaluate different factors that cause medical graduates to not publish their research. These factors may be related either to the individuals or to their medical institutions.

In this study, we attempted to interpret the reasons for noncompletion of research projects because the most important factor in postgraduate master’s degree programs at the Cairo University Faculty of Medicine is completion of a degree-related thesis for publication. We administered a web-based survey with several items to determine if a group of medical doctors completed their research projects and published them and to elucidate any difficulties they faced in publishing their research.

## Methods

### Data Collection

**Figure 1:**
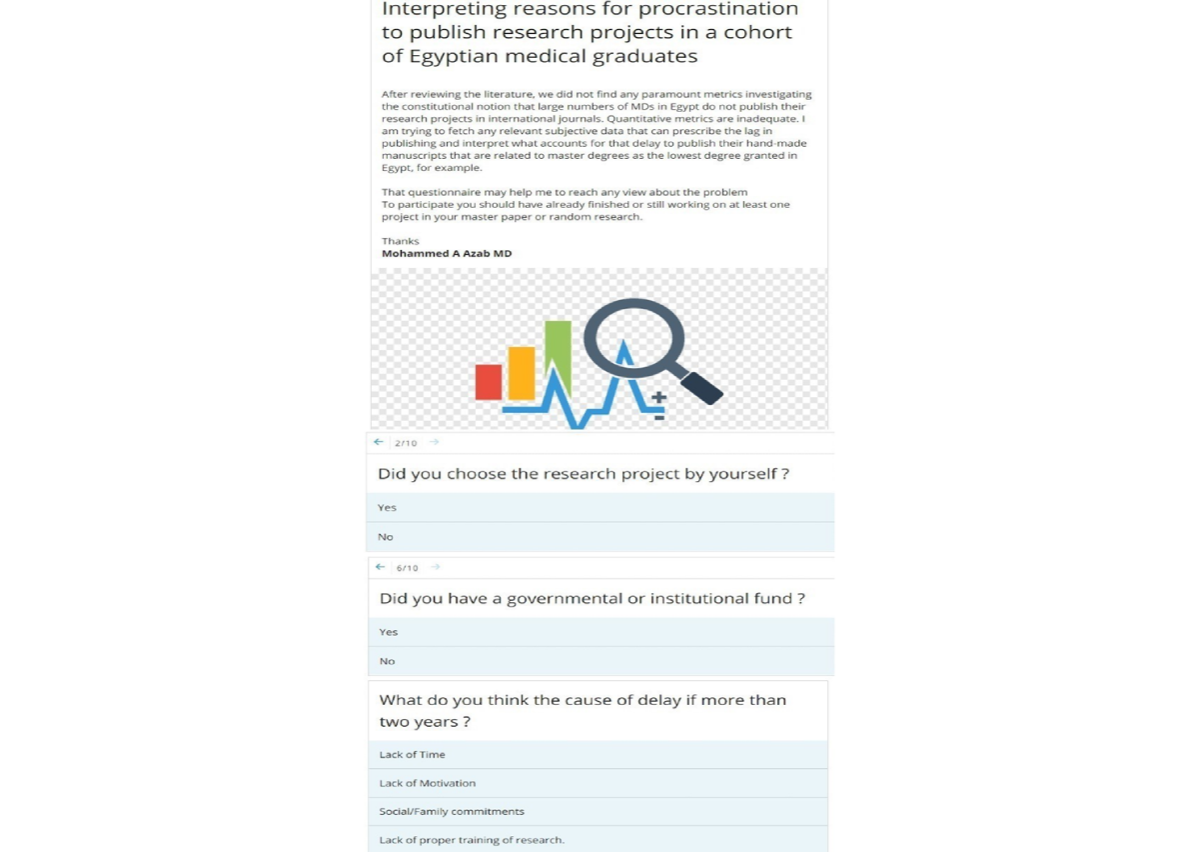
Screenshot of the web-based self-reported questionnaire administered to the study participants.

A self-reported web-based questionnaire survey that included 10 questions was introduced to a group of medical school graduates through Facebook and WhatsApp (Figure 1, Table 1). All respondents were Cairo University Faculty of Medicine medical graduates, and they specialized in different clinical and academic departments. The survey asked about the respondents’ interest in research, possible applications of their research project in the medical field, reasons for the delay in completion (ie, the delay in final publication), and suggested reasons for journal rejection. The research success of the participants was measured by the completion rate of publishing any number of papers, even one paper, as well as their state of authorship of the paper (first or other author). Out of 150 recipients, 130 responded to the survey in the period from June 15-30, 2020. Data from the survey were exported to an Excel spreadsheet (Microsoft Corporation) and analyzed using GraphPad Prism, version 8.0.0, for Windows (GraphPad Software Inc). We considered two-tailed *P* values <.05 to be statistically significant for all differences. Descriptive analysis was performed regarding age, gender, and marital status. The Mann-Whitney U test was applied as a nonparametric test to identify significant differences for each of the variables under study and how they relate to each other, such as whether the rates of completion and publishing differ according to the graduate’s marital status or the availability of a helpful mentor.

**Table 1:** Questions and possible responses in the web-based self-reported questionnaire.

Question	Responses
Are you interested in research, whether clinical or basic science?	Yes No
Did you choose your research project by yourself?	Yes No
Did you consider it a new addition to academic and clinical research?	Yes No
What do you think the cause of delay was if your research was delayed more than two years?	Lack of time Lack of motivation Social/family commitments Lack of proper training of research. Unsupportive supervisor Lack of proper internet facilities/opportunities Lack of proper laboratory facilities Lack of funding Overloaded curriculum
Did you have a governmental or institutional fund?	Yes No
Have you finished your research project?	Yes No
How long did it take from finishing the entire study to publishing it, if it has already been published?	2 years More than 2 years Didn’t publish
Do you think your supervisor was cooperative?	Yes No
If your work was not published, why do you think it was not published?	Not beneficial Discussed many times in the literature (“lack of novelty”) Plagiarism The manuscript is out of scope of the journal The peer reviewers’ comments were not properly answered or went unanswered Incomplete or insufficient information in the abstract Title was not representative of the study Inaccurate or inconsistent data were reported Defective tables or figures

### Ethics Approval and Consent to Participate

All participants were consented to share their views and test results in the survey. All data used were anonymized and encrypted to comply with all research ethics. All procedures performed in studies involving human participants were in accordance with the ethical standards of the institutional and/or national research committee and with the 1964 Helsinki declaration and its later amendments or comparable ethical standards.

## Results

Out of 150 graduates, 130 responded to the web-based survey. Frequencies were counted for demographic data such as age, gender, and marital status (Table 2). By analyzing the results, we found that 73/130 of the participants (56.2%) were not interested in research. Among the contributors, 102/130 (78.5%) did not secure any type of funding for their master's degree program research project.

**Table 2:** Demographic data of the study respondents (N=130).

Variable			Value
**Age (years)**			
	Range	23-38	
	Mean (SD)	29.13 (3.9)	
**Sex, n (%)**			
	Male	72 (55.4)	
	Female	58 (44.6)	
**Marital status, n (%)**			
	Married	76 (58.5)	
	Single	54 (41.5)	

Regarding the attitudes of individuals in different groups toward research facilities, we observed that (34/130 of them (26.2%) described their mentors as unsupportive and considered that lack of support to be a cause of delay in publishing their master’s degree research manuscripts. With further assessment of different factors that influenced the research activities, 39/130 participants (30%) reported that the lack of proper training played a principal role in their lack of interest in research; 34/130 (26.2%) complained about unsupportive research mentors as a cause of failing to finalize their assigned research project, while 26/130 (20%) declared that social commitments were a hurdle to their motivation to pursue their research projects (Table 3).

**Table 3:** Reasons for participants’ failure to complete their assigned research projects (N=130).

Reason	Value, n (%)
Lack of motivation	12 (9.2)
Uncooperative supervisor	34 (26.2)
Lack of proper training	39 (30)
Difficulty of finding a suitable research project	2 (1.5)
Social commitments	26 (20)
Lack of time	10 (7.7)
Other causes	7 (5.3)

A variety of factors that prevented the participants’ research projects from being published are summarized in Table 4. The most frequent proposed etiology was a lack of novel ideas, as most of the used research designs were discussed extensively in the literature and were not considered to be new ideas. Of the medical graduates in the study group, 21/130 (16.2%) judged their research to be “pointless.”

**Table 4:** Suggested reasons for rejection of submitted manuscripts among the study participants (N=130).

Reason	Value, n (%)
Discussed many times (lack of novelty)	39 (30)
Not beneficial	16 (12.3)
Plagiarism	14 (17.77)
Incomplete information in the abstract	13 (10)
Out of scope of the journal	10 (7.7)
Reviewers’ questions not answered properly	8 (6.15)

Using the Mann-Whitney *U* test to assess relationships and differences between available variables, we found that the frequency of noncompletion of master’s degree research projects was higher in married individuals than in those who were single, as testified by a two-tailed *P* value of .002. Moreover, we did not find any relationship between interest in research and the completion rate of the research project (*P*=.50, which is not significant). There is a strong relationship between the availability of a supportive research supervisor and the completion of a master’s degree project; of 130 students who completed their research projects, 96 stated they had a cooperative mentor (96/130, 73.8%; *P=*.04).

## Discussion

### Principal Findings

Postgraduate medical education is constantly changing on a large scientific scale. Very large data sets and technological advances have begun to demonstrate different clinical applications. Therefore, a proper methodology to investigate the lack of interest in research among medical graduates is needed. Research is a dynamic process that includes creating a research idea, followed by scientific writing and publishing. Some factors impede this process at any step; therefore, we attempted to summarize these factors as well as possible.

In this study, 72.3% of the participants reported that they did not have a good supervising mentor, and they considered that to be a cause of delay in publishing their research. Mentorship is a responsibility that requires diligent availability of time and scientific resources, and it is a crucial item in research implementation [2]. In developing countries, mentors are usually busy focusing on clinical activities such as performing surgeries and attending outpatient clinics, and they do not have sufficient time for research mentoring [2]. The significance of providing proper active mentorship at academic and clinical institutions is receiving increasing attention worldwide [3].

We observed that 56.2% of the respondents to our survey were not interested in research. Therefore, it is pivotal to delineate the factors that eventually lead to this lack of interest. One remarkable issue that may decrease interest in research is the absence of an interesting project idea. Scientific idea design is an essential step in the process of research accomplishment. Lack of novelty and outdated ideas are a major cause of manuscript rejection; in this study, this represents the cause for rejection of 39.2% of the participants (51/130), as analyzed above. The research question plays a valuable role in the overall research process and should be fashioned meticulously [4].

It is noteworthy that most research is costly and requires funding. Lack of adequate funding, especially in developing countries, is an obstacle that disrupts the process of publishing. Resource allocation for research is a critical point that should be well considered. In this study, 78.5% of medical graduates did not have any funding for their research projects. We found a strong relationship between the scarcity of funds and the noncompletion of research (*P*=.04). Financial aid for researchers at academic institutions should be assigned properly, as this is considered to be an investment in proper patient care in the future [5].

One of the factors that affected the completion rate was the social restrictions for married medical graduates. Of the 130 respondents, 76 (58.5%) were married, and we found that a significant number of married researchers did not complete or publish their work compared to unmarried researchers, as indicated by the *P* value of .002. Family commitments and social relations are considered to be a source of stress for some medical graduates, specifically at the beginning of their careers. The academic performance of married researchers will certainly improve if both partners are cooperative, are helpful, and support each other [6].

The rate of rejection of manuscripts submitted to different medical journals is higher in developing countries than in Europe and North America [7]. We attempted to explicate the causes of manuscript rejection and review the suggested reasons among a group of postgraduate medical researchers. We found that 51 of the 130 persons involved in the study (39.2%) suggested that their work was rejected mainly because the idea was extensively discussed in the literature and did not provide any new scientific or statistical data to the reader. Therefore, a careful, systematic selection of the idea of the research project should be emphasized. The next suggested reason is the lack of value of the research idea; 16% of the participating physicians reported that their research projects were not beneficial. Plagiarism was the cause of 13.8% of the rejected work in this study. Plagiarism is a serious ethical issue that is considered to be misconduct by most research institutions. It is important for research program directors to arrange workshops and lectures to teach young researchers how to avoid plagiarism. The Indian Journal of Dermatology prohibited a group of Tunisian researchers from publishing in the journal due to the submission of a plagiarized article [8]. Proper training and educational courses about scientific writing should be presented to medical students and graduates to improve their research skills, which will help them to accomplish their research projects for the sake of improving patient care.

### Limitations of the Study

Online questionnaire surveys are subjective and liable to bias from some respondents. The sample size is small, so it cannot be generalized; moreover, the study relates to only one institution in Egypt, the Cairo University Faculty of Medicine, and thus it does not represent all medical graduates. Generalization of the study requires a multi-institution approach.

### Conclusion

Factors affecting the pursuit of research by medical graduates are clear to the scientific community. Dedicated efforts and organized plans should be assigned to help medical graduates in developing countries to improve their research skills.
